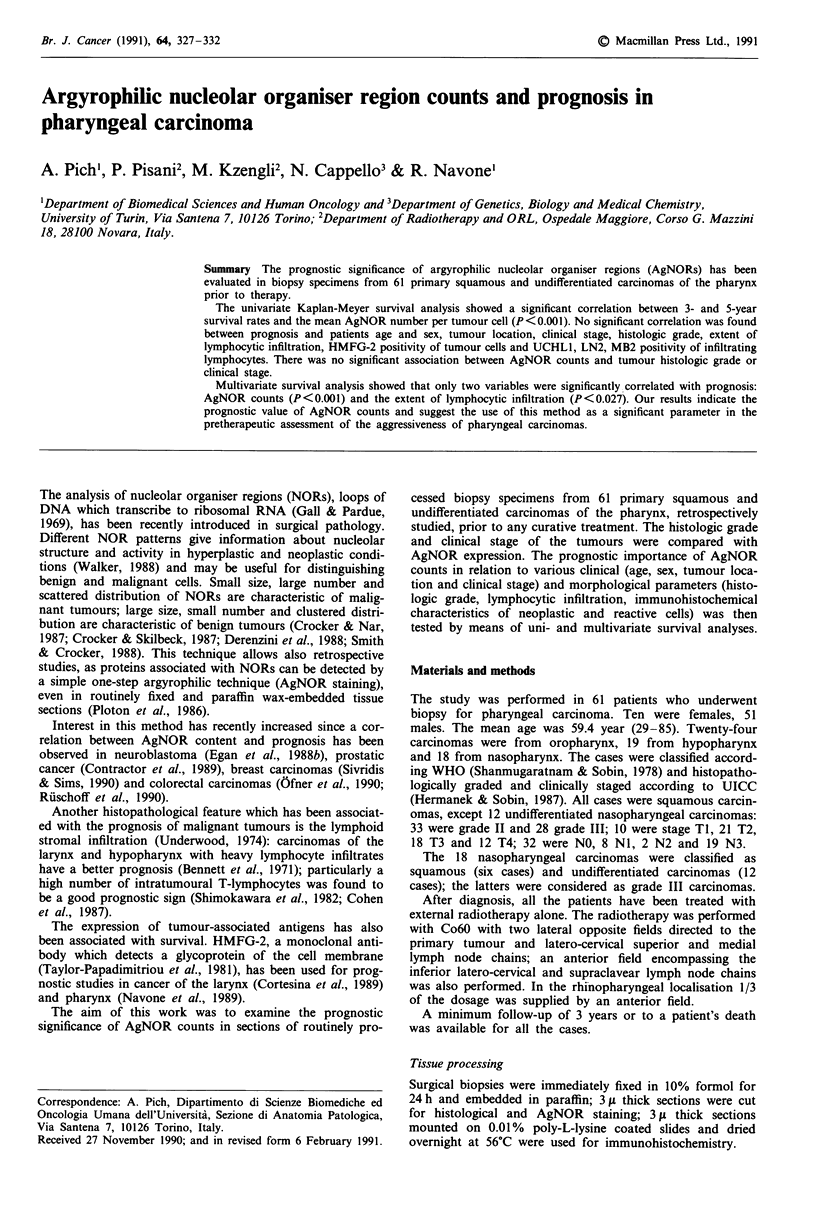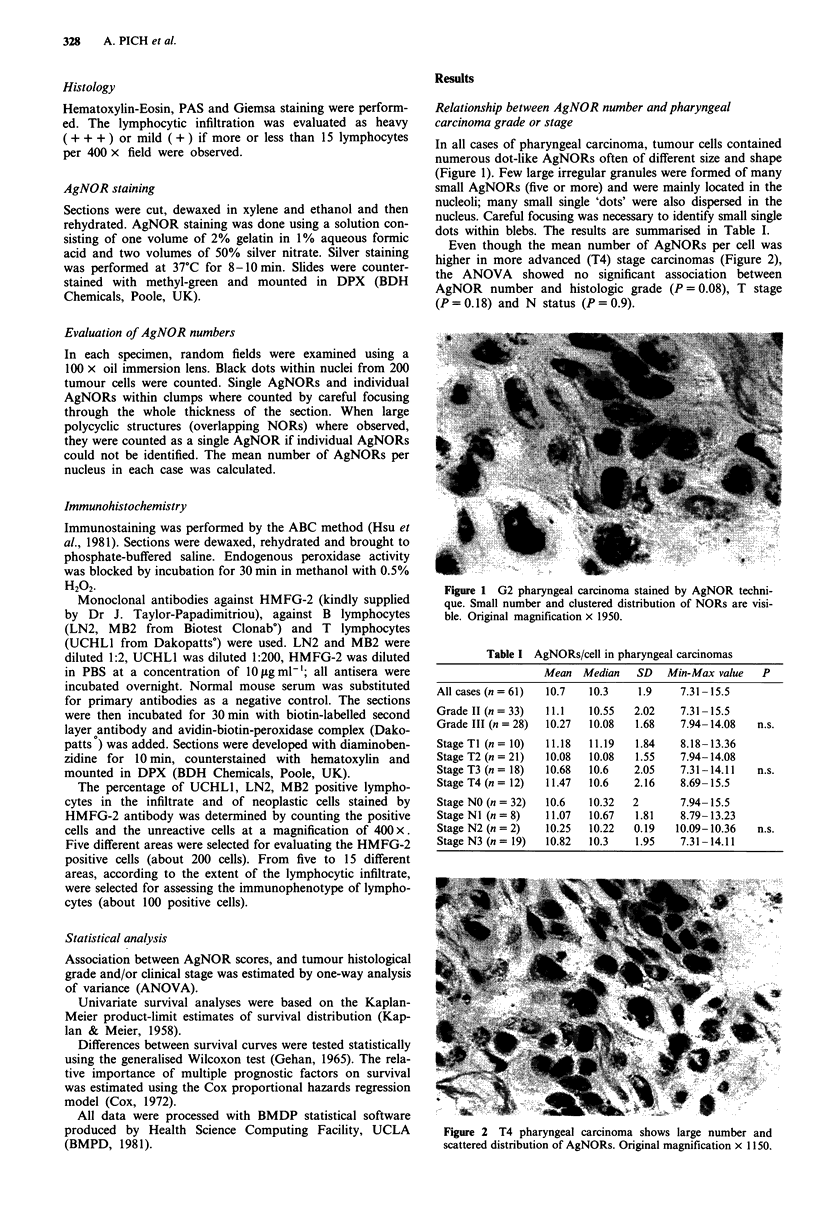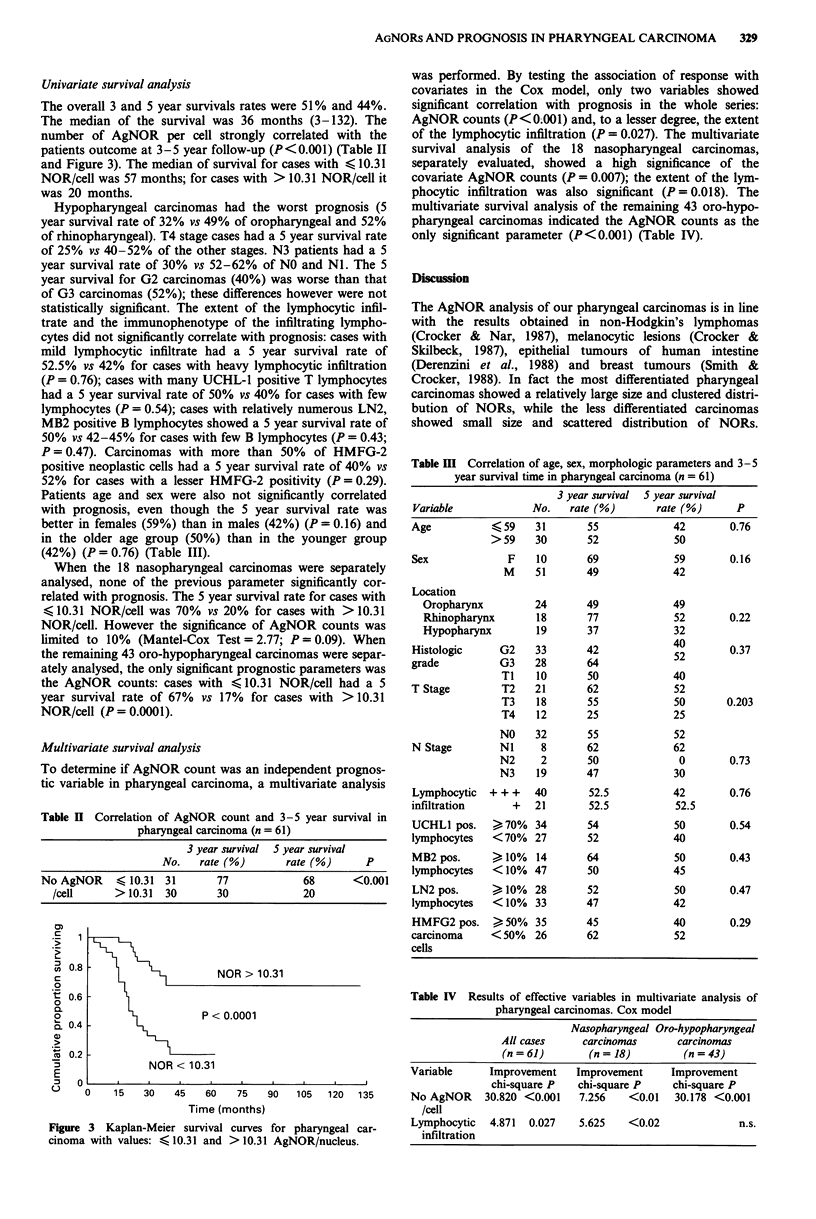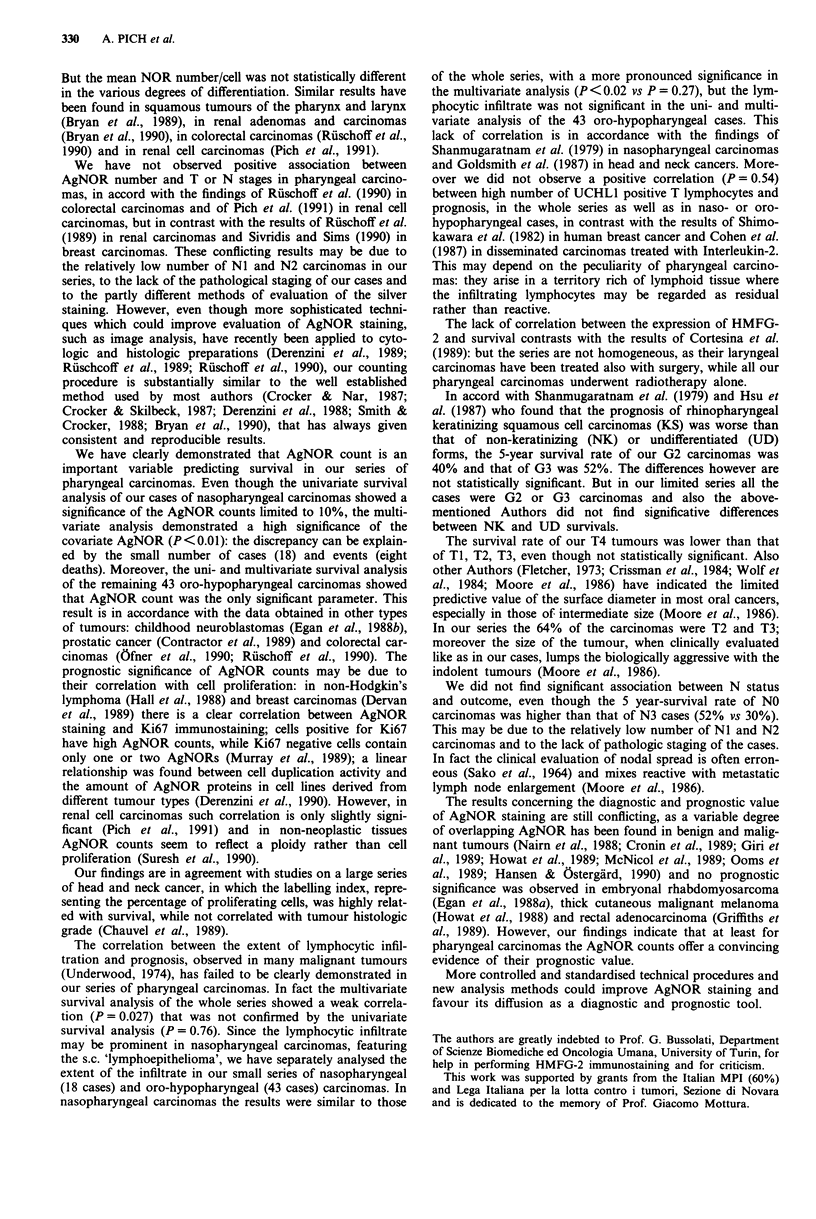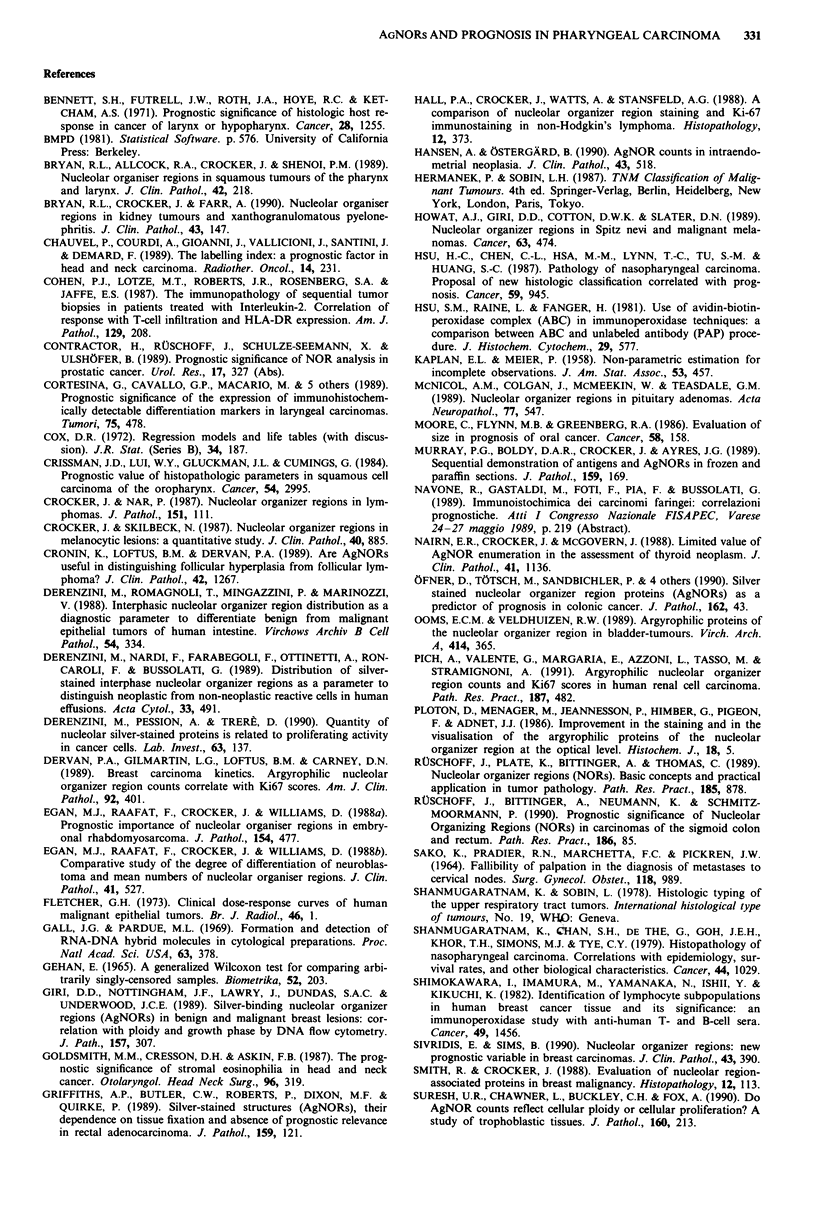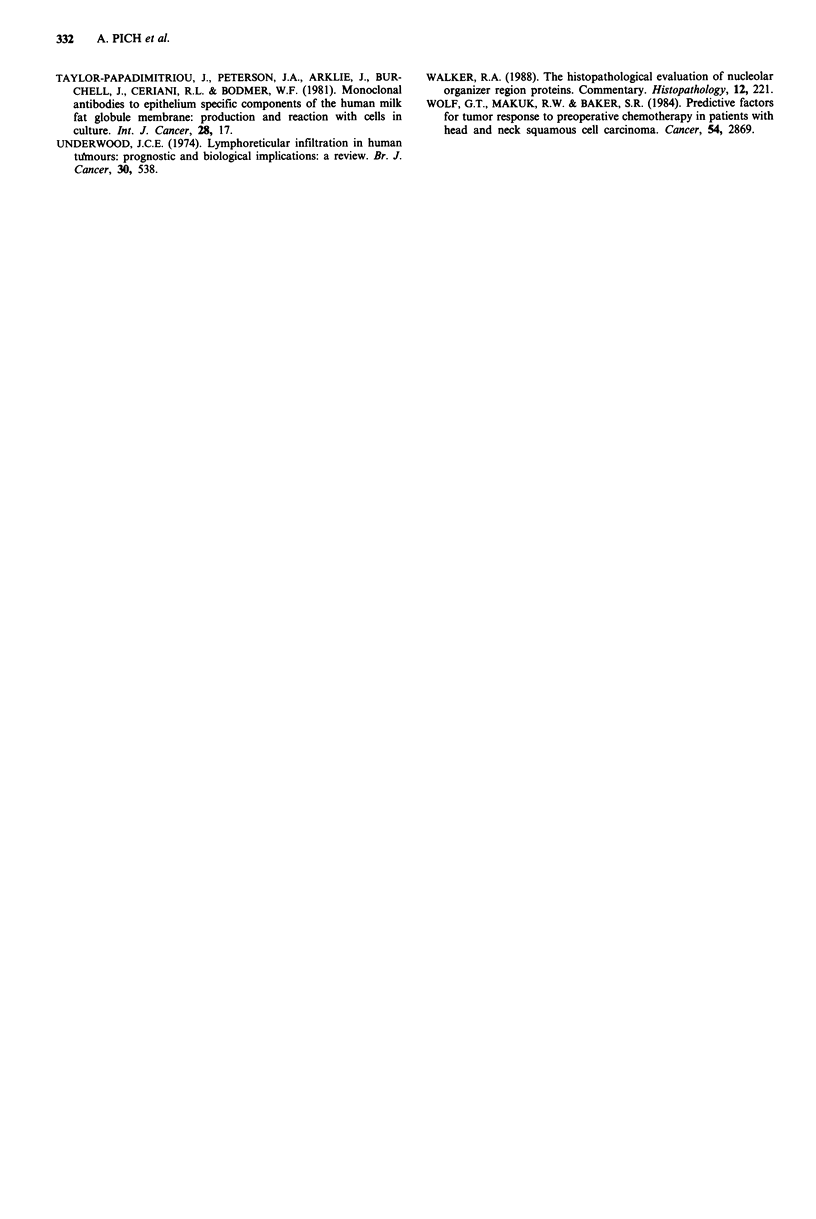# Argyrophilic nucleolar organiser region counts and prognosis in pharyngeal carcinoma.

**DOI:** 10.1038/bjc.1991.300

**Published:** 1991-08

**Authors:** A. Pich, P. Pisani, M. Kzengli, N. Cappello, R. Navone

**Affiliations:** Department of Biomedical Sciences and Human Oncology, University of Turin, Italy.

## Abstract

**Images:**


Br. J. Cancer (1991), 64, 327-332 © Macmillan Press Ltd., 1991

### Argyrophilic nucleolar organiser region counts and prognosis in pharyngeal carcinoma

A. Pich[1], P. Pisani[2], M. Kzengli[2], N. Cappello[3] & R. Navone[1]

[1]*Department of Biomedical Sciences and Human Oncology and* [3]*Department of Genetics, Biology and Medical Chemistry, University of Turin, Via Santena 7, 10126 Torino;* [2]*Department of Radiotherapy and ORL, Ospedale Maggiore, Corso G. Mazzini 18, 28100 Novara, Italy.*

**Summary** The prognostic significance of argyrophilic nucleolar organiser regions (AgNORs) has been evaluated in biopsy specimens from 61 primary squamous and undifferentiated carcinomas of the pharynx prior to therapy.

The univariate Kaplan-Meyer survival analysis showed a significant correlation between 3- and 5-year survival rates and the mean AgNOR number per tumour cell ($P<0.001$). No significant correlation was found between prognosis and patients age and sex, tumour location, clinical stage, histologic grade, extent of lymphocytic infiltration, HMFG-2 positivity of tumour cells and UCHL1, LN2, MB2 positivity of infiltrating lymphocytes. There was no significant association between AgNOR counts and tumour histologic grade or clinical stage.

Multivariate survival analysis showed that only two variables were significantly correlated with prognosis: AgNOR counts ($P<0.001$) and the extent of lymphocytic infiltration ($P<0.027$). Our results indicate the prognostic value of AgNOR counts and suggest the use of this method as a significant parameter in the pretherapeutic assessment of the aggressiveness of pharyngeal carcinomas.

The analysis of nucleolar organiser regions (NORs), loops of DNA which transcribe to ribosomal RNA (Gall & Pardue, 1969), has been recently introduced in surgical pathology. Different NOR patterns give information about nucleolar structure and activity in hyperplastic and neoplastic conditions (Walker, 1988) and may be useful for distinguishing benign and malignant cells. Small size, large number and scattered distribution of NORs are characteristic of malignant tumours; large size, small number and clustered distribution are characteristic of benign tumours (Crocker & Nar, 1987; Crocker & Skilbeck, 1987; Derenzini *et al.*, 1988; Smith & Crocker, 1988). This technique allows also retrospective studies, as proteins associated with NORs can be detected by a simple one-step argyrophilic technique (AgNOR staining), even in routinely fixed and paraffin wax-embedded tissue sections (Ploton *et al.*, 1986).

Interest in this method has recently increased since a correlation between AgNOR content and prognosis has been observed in neuroblastoma (Egan *et al.*, 1988*b*), prostatic cancer (Contractor *et al.*, 1989), breast carcinomas (Sivridis & Sims, 1990) and colorectal carcinomas (Öfner *et al.*, 1990; Rüschoff *et al.*, 1990).

Another histopathological feature which has been associated with the prognosis of malignant tumours is the lymphoid stromal infiltration (Underwood, 1974): carcinomas of the larynx and hypopharynx with heavy lymphocyte infiltrates have a better prognosis (Bennett *et al.*, 1971); particularly a high number of intratumoural T-lymphocytes was found to be a good prognostic sign (Shimokawara *et al.*, 1982; Cohen *et al.*, 1987).

The expression of tumour-associated antigens has also been associated with survival. HMFG-2, a monoclonal antibody which detects a glycoprotein of the cell membrane (Taylor-Papadimitriou *et al.*, 1981), has been used for prognostic studies in cancer of the larynx (Cortesina *et al.*, 1989) and pharynx (Navone *et al.*, 1989).

The aim of this work was to examine the prognostic significance of AgNOR counts in sections of routinely processed biopsy specimens from 61 primary squamous and undifferentiated carcinomas of the pharynx, retrospectively studied, prior to any curative treatment. The histologic grade and clinical stage of the tumours were compared with AgNOR expression. The prognostic importance of AgNOR counts in relation to various clinical (age, sex, tumour location and clinical stage) and morphological parameters (histologic grade, lymphocytic infiltration, immunohistochemical characteristics of neoplastic and reactive cells) was then tested by means of uni- and multivariate survival analyses.

### Materials and methods

The study was performed in 61 patients who underwent biopsy for pharyngeal carcinoma. Ten were females, 51 males. The mean age was 59.4 year (29–85). Twenty-four carcinomas were from oropharynx, 19 from hypopharynx and 18 from nasopharynx. The cases were classified according WHO (Shanmugaratnam & Sobin, 1978) and histopathologically graded and clinically staged according to UICC (Hermanek & Sobin, 1987). All cases were squamous carcinomas, except 12 undifferentiated nasopharyngeal carcinomas: 33 were grade II and 28 grade III; 10 were stage T1, 21 T2, 18 T3 and 12 T4; 32 were N0, 8 N1, 2 N2 and 19 N3.

The 18 nasopharyngeal carcinomas were classified as squamous (six cases) and undifferentiated carcinomas (12 cases); the latters were considered as grade III carcinomas.

After diagnosis, all the patients have been treated with external radiotherapy alone. The radiotherapy was performed with Co60 with two lateral opposite fields directed to the primary tumour and latero-cervical superior and medial lymph node chains; an anterior field encompassing the inferior latero-cervical and supraclavear lymph node chains was also performed. In the rhinopharyngeal localisation 1/3 of the dosage was supplied by an anterior field.

A minimum follow-up of 3 years or to a patient's death was available for all the cases.

### *Tissue processing*

Surgical biopsies were immediately fixed in 10% formol for 24 h and embedded in paraffin; 3 μ thick sections were cut for histological and AgNOR staining; 3 μ thick sections mounted on 0.01% poly-L-lysine coated slides and dried overnight at 56°C were used for immunohistochemistry.

Correspondence: A. Pich, Dipartimento di Scienze Biomediche ed Oncologia Umana dell'Università, Sezione di Anatomia Patologica, Via Santena 7, 10126 Torino, Italy.
Received 27 November 1990; and in revised form 6 February 1991.

### Histology

Hematoxylin-Eosin, PAS and Giemsa staining were performed. The lymphocytic infiltration was evaluated as heavy (+++) or mild (+) if more or less than 15 lymphocytes per 400 × field were observed.

### AgNOR staining

Sections were cut, dewaxed in xylene and ethanol and then rehydrated. AgNOR staining was done using a solution consisting of one volume of 2% gelatin in 1% aqueous formic acid and two volumes of 50% silver nitrate. Silver staining was performed at 37°C for 8–10 min. Slides were counterstained with methyl-green and mounted in DPX (BDH Chemicals, Poole, UK).

### Evaluation of AgNOR numbers

In each specimen, random fields were examined using a 100 × oil immersion lens. Black dots within nuclei from 200 tumour cells were counted. Single AgNORs and individual AgNORs within clumps where counted by careful focusing through the whole thickness of the section. When large polycyclic structures (overlapping NORs) where observed, they were counted as a single AgNOR if individual AgNORs could not be identified. The mean number of AgNORs per nucleus in each case was calculated.

### Immunohistochemistry

Immunostaining was performed by the ABC method (Hsu et al., 1981). Sections were dewaxed, rehydrated and brought to phosphate-buffered saline. Endogenous peroxidase activity was blocked by incubation for 30 min in methanol with 0.5% $H_2O_2$.

Monoclonal antibodies against HMFG-2 (kindly supplied by Dr J. Taylor-Papadimitriou), against B lymphocytes (LN2, MB2 from Biotest Clonab°) and T lymphocytes (UCHL1 from Dakopatts°) were used. LN2 and MB2 were diluted 1:2, UCHL1 was diluted 1:200, HMFG-2 was diluted in PBS at a concentration of $10 \mu g ml^{-1}$; all antisera were incubated overnight. Normal mouse serum was substituted for primary antibodies as a negative control. The sections were then incubated for 30 min with biotin-labelled second layer antibody and avidin-biotin-peroxidase complex (Dakopatts°) was added. Sections were developed with diaminobenzidine for 10 min, counterstained with hematoxylin and mounted in DPX (BDH Chemicals, Poole, UK).

The percentage of UCHL1, LN2, MB2 positive lymphocytes in the infiltrate and of neoplastic cells stained by HMFG-2 antibody was determined by counting the positive cells and the unreactive cells at a magnification of 400×. Five different areas were selected for evaluating the HMFG-2 positive cells (about 200 cells). From five to 15 different areas, according to the extent of the lymphocytic infiltrate, were selected for assessing the immunophenotype of lymphocytes (about 100 positive cells).

### Statistical analysis

Association between AgNOR scores, and tumour histological grade and/or clinical stage was estimated by one-way analysis of variance (ANOVA).

Univariate survival analyses were based on the Kaplan-Meier product-limit estimates of survival distribution (Kaplan & Meier, 1958).

Differences between survival curves were tested statistically using the generalised Wilcoxon test (Gehan, 1965). The relative importance of multiple prognostic factors on survival was estimated using the Cox proportional hazards regression model (Cox, 1972).

All data were processed with BMDP statistical software produced by Health Science Computing Facility, UCLA (BMPD, 1981).

### Results

### Relationship between AgNOR number and pharyngeal carcinoma grade or stage

In all cases of pharyngeal carcinoma, tumour cells contained numerous dot-like AgNORs often of different size and shape (Figure 1). Few large irregular granules were formed of many small AgNORs (five or more) and were mainly located in the nucleoli; many small single 'dots' were also dispersed in the nucleus. Careful focusing was necessary to identify small single dots within blebs. The results are summarised in Table I.

Even though the mean number of AgNORs per cell was higher in more advanced (T4) stage carcinomas (Figure 2), the ANOVA showed no significant association between AgNOR number and histologic grade ($P = 0.08$), T stage ($P = 0.18$) and N status ($P = 0.9$).

**Figure 1** G2 pharyngeal carcinoma stained by AgNOR technique. Small number and clustered distribution of NORs are visible. Original magnification × 1950.

**Table I** AgNORs/cell in pharyngeal carcinomas

| | Mean | Median | SD | Min-Max value | P |
|---|---|---|---|---|---|
| All cases ($n = 61$) | 10.7 | 10.3 | 1.9 | 7.31–15.5 | |
| Grade II ($n = 33$) | 11.1 | 10.55 | 2.02 | 7.31–15.5 | |
| Grade III ($n = 28$) | 10.27 | 10.08 | 1.68 | 7.94–14.08 | n.s. |
| Stage T1 ($n = 10$) | 11.18 | 11.19 | 1.84 | 8.18–13.36 | |
| Stage T2 ($n = 21$) | 10.08 | 10.08 | 1.55 | 7.94–14.08 | |
| Stage T3 ($n = 18$) | 10.68 | 10.6 | 2.05 | 7.31–14.11 | n.s. |
| Stage T4 ($n = 12$) | 11.47 | 10.6 | 2.16 | 8.69–15.5 | |
| Stage N0 ($n = 32$) | 10.6 | 10.32 | 2 | 7.94–15.5 | |
| Stage N1 ($n = 8$) | 11.07 | 10.67 | 1.81 | 8.79–13.23 | |
| Stage N2 ($n = 2$) | 10.25 | 10.22 | 0.19 | 10.09–10.36 | n.s. |
| Stage N3 ($n = 19$) | 10.82 | 10.3 | 1.95 | 7.31–14.11 | |

**Figure 2** T4 pharyngeal carcinoma shows large number and scattered distribution of AgNORs. Original magnification × 1150.

*Univariate survival analysis*

The overall 3 and 5 year survivals rates were 51% and 44%. The median of the survival was 36 months (3–132). The number of AgNOR per cell strongly correlated with the patients outcome at 3–5 year follow-up ($P<0.001$) (Table II and Figure 3). The median of survival for cases with $\leqslant$10.31 NOR/cell was 57 months; for cases with $>$10.31 NOR/cell it was 20 months.

Hypopharyngeal carcinomas had the worst prognosis (5 year survival rate of 32% *vs* 49% of oropharyngeal and 52% of rhinopharyngeal). T4 stage cases had a 5 year survival rate of 25% *vs* 40–52% of the other stages. N3 patients had a 5 year survival rate of 30% *vs* 52–62% of N0 and N1. The 5 year survival for G2 carcinomas (40%) was worse than that of G3 carcinomas (52%); these differences however were not statistically significant. The extent of the lymphocytic infiltrate and the immunophenotype of the infiltrating lymphocytes did not significantly correlate with prognosis: cases with mild lymphocytic infiltrate had a 5 year survival rate of 52.5% *vs* 42% for cases with heavy lymphocytic infiltration ($P=0.76$); cases with many UCHL-1 positive T lymphocytes had a 5 year survival rate of 50% *vs* 40% for cases with few lymphocytes ($P=0.54$); cases with relatively numerous LN2, MB2 positive B lymphocytes showed a 5 year survival rate of 50% *vs* 42–45% for cases with few B lymphocytes ($P=0.43$; $P=0.47$). Carcinomas with more than 50% of HMFG-2 positive neoplastic cells had a 5 year survival rate of 40% *vs* 52% for cases with a lesser HMFG-2 positivity ($P=0.29$). Patients age and sex were also not significantly correlated with prognosis, even though the 5 year survival rate was better in females (59%) than in males (42%) ($P=0.16$) and in the older age group (50%) than in the younger group (42%) ($P=0.76$) (Table III).

When the 18 nasopharyngeal carcinomas were separately analysed, none of the previous parameter significantly correlated with prognosis. The 5 year survival rate for cases with $\leqslant$10.31 NOR/cell was 70% *vs* 20% for cases with $>$10.31 NOR/cell. However the significance of AgNOR counts was limited to 10% (Mantel-Cox Test = 2.77; $P=0.09$). When the remaining 43 oro-hypopharyngeal carcinomas were separately analysed, the only significant prognostic parameters was the AgNOR counts: cases with $\leqslant$10.31 NOR/cell had a 5 year survival rate of 67% *vs* 17% for cases with $>$10.31 NOR/cell ($P=0.0001$).

*Multivariate survival analysis*

To determine if AgNOR count was an independent prognostic variable in pharyngeal carcinoma, a multivariate analysis was performed. By testing the association of response with covariates in the Cox model, only two variables showed significant correlation with prognosis in the whole series: AgNOR counts ($P<0.001$) and, to a lesser degree, the extent of the lymphocytic infiltration ($P=0.027$). The multivariate survival analysis of the 18 nasopharyngeal carcinomas, separately evaluated, showed a high significance of the covariate AgNOR counts ($P=0.007$); the extent of the lymphocytic infiltration was also significant ($P=0.018$). The multivariate survival analysis of the remaining 43 oro-hypopharyngeal carcinomas indicated the AgNOR counts as the only significant parameter ($P<0.001$) (Table IV).

**Table II** Correlation of AgNOR count and 3–5 year survival in pharyngeal carcinoma ($n=61$)

| | | No. | *3 year survival rate (%)* | *5 year survival rate (%)* | *P* |
|---|---|---|---|---|---|
| No AgNOR /cell | $\leqslant$10.31 | 31 | 77 | 68 | <0.001 |
| | $>$10.31 | 30 | 30 | 20 | |

**Figure 3** Kaplan-Meier survival curves for pharyngeal carcinoma with values: $\leqslant$10.31 and $>$10.31 AgNOR/nucleus.

### Discussion

The AgNOR analysis of our pharyngeal carcinomas is in line with the results obtained in non-Hodgkin's lymphomas (Crocker & Nar, 1987), melanocytic lesions (Crocker & Skilbeck, 1987), epithelial tumours of human intestine (Derenzini *et al.*, 1988) and breast tumours (Smith & Crocker, 1988). In fact the most differentiated pharyngeal carcinomas showed a relatively large size and clustered distribution of NORs, while the less differentiated carcinomas showed small size and scattered distribution of NORs.

**Table III** Correlation of age, sex, morphologic parameters and 3–5 year survival time in pharyngeal carcinoma ($n=61$)

| *Variable* | | *No.* | *3 year survival rate (%)* | *5 year survival rate (%)* | *P* |
|---|---|---|---|---|---|
| Age | $\leqslant$59 | 31 | 55 | 42 | 0.76 |
| | $>$59 | 30 | 52 | 50 | |
| Sex | F | 10 | 69 | 59 | 0.16 |
| | M | 51 | 49 | 42 | |
| Location | | | | | |
| Oropharynx | | 24 | 49 | 49 | |
| Rhinopharynx | | 18 | 77 | 52 | 0.22 |
| Hypopharynx | | 19 | 37 | 32 | |
| Histologic grade | G2 | 33 | 42 | 40 | 0.37 |
| | G3 | 28 | 64 | 52 | |
| T Stage | T1 | 10 | 50 | 40 | |
| | T2 | 21 | 62 | 52 | |
| | T3 | 18 | 55 | 50 | 0.203 |
| | T4 | 12 | 25 | 25 | |
| N Stage | N0 | 32 | 55 | 52 | |
| | N1 | 8 | 62 | 62 | |
| | N2 | 2 | 50 | 0 | 0.73 |
| | N3 | 19 | 47 | 30 | |
| Lymphocytic infiltration | + + + | 40 | 52.5 | 42 | 0.76 |
| | + | 21 | 52.5 | 52.5 | |
| UCHL1 pos. lymphocytes | $\geqslant$70% | 34 | 54 | 50 | 0.54 |
| | $<$70% | 27 | 52 | 40 | |
| MB2 pos. lymphocytes | $\geqslant$10% | 14 | 64 | 50 | 0.43 |
| | $<$10% | 47 | 50 | 45 | |
| LN2 pos. lymphocytes | $\geqslant$10% | 28 | 52 | 50 | 0.47 |
| | $<$10% | 33 | 47 | 42 | |
| HMFG2 pos. carcinoma cells | $\geqslant$50% | 35 | 45 | 40 | 0.29 |
| | $<$50% | 26 | 62 | 52 | |

**Table IV** Results of effective variables in multivariate analysis of pharyngeal carcinomas. Cox model

| | *All cases (n = 61)* | | *Nasopharyngeal carcinomas (n = 18)* | | *Oro-hypopharyngeal carcinomas (n = 43)* | |
|---|---|---|---|---|---|---|
| Variable | Improvement chi-square | P | Improvement chi-square | P | Improvement chi-square | P |
| No AgNOR /cell | 30.820 | <0.001 | 7.256 | <0.01 | 30.178 | <0.001 |
| Lymphocytic infiltration | 4.871 | 0.027 | 5.625 | <0.02 | | n.s. |

But the mean NOR number/cell was not statistically different in the various degrees of differentiation. Similar results have been found in squamous tumours of the pharynx and larynx (Bryan *et al.*, 1989), in renal adenomas and carcinomas (Bryan *et al.*, 1990), in colorectal carcinomas (Rüschoff *et al.*, 1990) and in renal cell carcinomas (Pich *et al.*, 1991).

We have not observed positive association between AgNOR number and T or N stages in pharyngeal carcinomas, in accord with the findings of Rüschoff *et al.* (1990) in colorectal carcinomas and of Pich *et al.* (1991) in renal cell carcinomas, but in contrast with the results of Rüschoff *et al.* (1989) in renal carcinomas and Sivridis and Sims (1990) in breast carcinomas. These conflicting results may be due to the relatively low number of N1 and N2 carcinomas in our series, to the lack of the pathological staging of our cases and to the partly different methods of evaluation of the silver staining. However, even though more sophisticated techniques which could improve evaluation of AgNOR staining, such as image analysis, have recently been applied to cytologic and histologic preparations (Derenzini *et al.*, 1989; Rüschcoff *et al.*, 1989; Rüschoff *et al.*, 1990), our counting procedure is substantially similar to the well established method used by most authors (Crocker & Nar, 1987; Crocker & Skilbeck, 1987; Derenzini *et al.*, 1988; Smith & Crocker, 1988; Bryan *et al.*, 1990), that has always given consistent and reproducible results.

We have clearly demonstrated that AgNOR count is an important variable predicting survival in our series of pharyngeal carcinomas. Even though the univariate survival analysis of our cases of nasopharyngeal carcinomas showed a significance of the AgNOR counts limited to 10%, the multivariate analysis demonstrated a high significance of the covariate AgNOR ($P < 0.01$): the discrepancy can be explained by the small number of cases (18) and events (eight deaths). Moreover, the uni- and multivariate survival analysis of the remaining 43 oro-hypopharyngeal carcinomas showed that AgNOR count was the only significant parameter. This result is in accordance with the data obtained in other types of tumours: childhood neuroblastomas (Egan *et al.*, 1988*b*), prostatic cancer (Contractor *et al.*, 1989) and colorectal carcinomas (Öfner *et al.*, 1990; Rüschoff *et al.*, 1990). The prognostic significance of AgNOR counts may be due to their correlation with cell proliferation: in non-Hodgkin's lymphoma (Hall *et al.*, 1988) and breast carcinomas (Dervan *et al.*, 1989) there is a clear correlation between AgNOR staining and Ki67 immunostaining; cells positive for Ki67 have high AgNOR counts, while Ki67 negative cells contain only one or two AgNORs (Murray *et al.*, 1989); a linear relationship was found between cell duplication activity and the amount of AgNOR proteins in cell lines derived from different tumour types (Derenzini *et al.*, 1990). However, in renal cell carcinomas such correlation is only slightly significant (Pich *et al.*, 1991) and in non-neoplastic tissues AgNOR counts seem to reflect a ploidy rather than cell proliferation (Suresh *et al.*, 1990).

Our findings are in agreement with studies on a large series of head and neck cancer, in which the labelling index, representing the percentage of proliferating cells, was highly related with survival, while not correlated with tumour histologic grade (Chauvel *et al.*, 1989).

The correlation between the extent of lymphocytic infiltration and prognosis, observed in many malignant tumours (Underwood, 1974), has failed to be clearly demonstrated in our series of pharyngeal carcinomas. In fact the multivariate survival analysis of the whole series showed a weak correlation ($P = 0.027$) that was not confirmed by the univariate survival analysis ($P = 0.76$). Since the lymphocytic infiltrate may be prominent in nasopharyngeal carcinomas, featuring the s.c. 'lymphoepithelioma', we have separately analysed the extent of the infiltrate in our small series of nasopharyngeal (18 cases) and oro-hypopharyngeal (43 cases) carcinomas. In nasopharyngeal carcinomas the results were similar to those of the whole series, with a more pronounced significance in the multivariate analysis ($P < 0.02$ *vs* $P = 0.27$), but the lymphocytic infiltrate was not significant in the uni- and multivariate analysis of the 43 oro-hypopharyngeal cases. This lack of correlation is in accordance with the findings of Shanmugaratnam *et al.* (1979) in nasopharyngeal carcinomas and Goldsmith *et al.* (1987) in head and neck cancers. Moreover we did not observe a positive correlation ($P = 0.54$) between high number of UCHL1 positive T lymphocytes and prognosis, in the whole series as well as in naso- or oro-hypopharyngeal cases, in contrast with the results of Shimokawara *et al.* (1982) in human breast cancer and Cohen *et al.* (1987) in disseminated carcinomas treated with Interleukin-2. This may depend on the peculiarity of pharyngeal carcinomas: they arise in a territory rich of lymphoid tissue where the infiltrating lymphocytes may be regarded as residual rather than reactive.

The lack of correlation between the expression of HMFG-2 and survival contrasts with the results of Cortesina *et al.* (1989): but the series are not homogeneous, as their laryngeal carcinomas have been treated also with surgery, while all our pharyngeal carcinomas underwent radiotherapy alone.

In accord with Shanmugaratnam *et al.* (1979) and Hsu *et al.* (1987) who found that the prognosis of rhinopharyngeal keratinizing squamous cell carcinomas (KS) was worse than that of non-keratinizing (NK) or undifferentiated (UD) forms, the 5-year survival rate of our G2 carcinomas was 40% and that of G3 was 52%. The differences however are not statistically significant. But in our limited series all the cases were G2 or G3 carcinomas and also the above-mentioned Authors did not find significative differences between NK and UD survivals.

The survival rate of our T4 tumours was lower than that of T1, T2, T3, even though not statistically significant. Also other Authors (Fletcher, 1973; Crissman *et al.*, 1984; Wolf *et al.*, 1984; Moore *et al.*, 1986) have indicated the limited predictive value of the surface diameter in most oral cancers, especially in those of intermediate size (Moore *et al.*, 1986). In our series the 64% of the carcinomas were T2 and T3; moreover the size of the tumour, when clinically evaluated like as in our cases, lumps the biologically aggressive with the indolent tumours (Moore *et al.*, 1986).

We did not find significant association between N status and outcome, even though the 5 year-survival rate of N0 carcinomas was higher than that of N3 cases (52% *vs* 30%). This may be due to the relatively low number of N1 and N2 carcinomas and to the lack of pathologic staging of the cases. In fact the clinical evaluation of nodal spread is often erroneous (Sako *et al.*, 1964) and mixes reactive with metastatic lymph node enlargement (Moore *et al.*, 1986).

The results concerning the diagnostic and prognostic value of AgNOR staining are still conflicting, as a variable degree of overlapping AgNOR has been found in benign and malignant tumours (Nairn *et al.*, 1988; Cronin *et al.*, 1989; Giri *et al.*, 1989; Howat *et al.*, 1989; McNicol *et al.*, 1989; Ooms *et al.*, 1989; Hansen & Östergärd, 1990) and no prognostic significance was observed in embryonal rhabdomyosarcoma (Egan *et al.*, 1988*a*), thick cutaneous malignant melanoma (Howat *et al.*, 1988) and rectal adenocarcinoma (Griffiths *et al.*, 1989). However, our findings indicate that at least for pharyngeal carcinomas the AgNOR counts offer a convincing evidence of their prognostic value.

More controlled and standardised technical procedures and new analysis methods could improve AgNOR staining and favour its diffusion as a diagnostic and prognostic tool.

The authors are greatly indebted to Prof. G. Bussolati, Department of Scienze Biomediche ed Oncologia Umana, University of Turin, for help in performing HMFG-2 immunostaining and for criticism.

This work was supported by grants from the Italian MPI (60%) and Lega Italiana per la lotta contro i tumori, Sezione di Novara and is dedicated to the memory of Prof. Giacomo Mottura.

### References

BENNETT, S.H., FUTRELL, J.W., ROTH, J.A., HOYE, R.C. & KETCHAM, A.S. (1971). Prognostic significance of histologic host response in cancer of larynx or hypopharynx. *Cancer*, **28**, 1255.

BMPD (1981). *Statistical Software*. p. 576. University of California Press: Berkeley.

BRYAN, R.L., ALLCOCK, R.A., CROCKER, J. & SHENOI, P.M. (1989). Nucleolar organiser regions in squamous tumours of the pharynx and larynx. *J. Clin. Pathol.*, **42**, 218.

BRYAN, R.L., CROCKER, J. & FARR, A. (1990). Nucleolar organiser regions in kidney tumours and xanthogranulomatous pyelonephritis. *J. Clin. Pathol.*, **43**, 147.

CHAUVEL, P., COURDI, A., GIOANNI, J., VALLICIONI, J., SANTINI, J. & DEMARD, F. (1989). The labelling index: a prognostic factor in head and neck carcinoma. *Radiother. Oncol.*, **14**, 231.

COHEN, P.J., LOTZE, M.T., ROBERTS, J.R., ROSENBERG, S.A. & JAFFE, E.S. (1987). The immunopathology of sequential tumor biopsies in patients treated with Interleukin-2. Correlation of response with T-cell infiltration and HLA-DR expression. *Am. J. Pathol.*, **129**, 208.

CONTRACTOR, H., RÜSCHOFF, J., SCHULZE-SEEMANN, X. & ULSHÖFER, B. (1989). Prognostic significance of NOR analysis in prostatic cancer. *Urol. Res.*, **17**, 327 (Abs).

CORTESINA, G., CAVALLO, G.P., MACARIO, M. & 5 others (1989). Prognostic significance of the expression of immunohistochemically detectable differentiation markers in laryngeal carcinomas. *Tumori*, **75**, 478.

COX, D.R. (1972). Regression models and life tables (with discussion). *J.R. Stat.* (Series B), **34**, 187.

CRISSMAN, J.D., LUI, W.Y., GLUCKMAN, J.L. & CUMINGS, G. (1984). Prognostic value of histopathologic parameters in squamous cell carcinoma of the oropharynx. *Cancer*, **54**, 2995.

CROCKER, J. & NAR, P. (1987). Nucleolar organizer regions in lymphomas. *J. Pathol.*, **151**, 111.

CROCKER, J. & SKILBECK, N. (1987). Nucleolar organizer regions in melanocytic lesions: a quantitative study. *J. Clin. Pathol.*, **40**, 885.

CRONIN, K., LOFTUS, B.M. & DERVAN, P.A. (1989). Are AgNORs useful in distinguishing follicular hyperplasia from follicular lymphoma? *J. Clin. Pathol.*, **42**, 1267.

DERENZINI, M., ROMAGNOLI, T., MINGAZZINI, P. & MARINOZZI, V. (1988). Interphasic nucleolar organizer region distribution as a diagnostic parameter to differentiate benign from malignant epithelial tumors of human intestine. *Virchows Archiv B Cell Pathol.*, **54**, 334.

DERENZINI, M., NARDI, F., FARABEGOLI, F., OTTINETTI, A., RONCAROLI, F. & BUSSOLATI, G. (1989). Distribution of silver-stained interphase nucleolar organizer regions as a parameter to distinguish neoplastic from non-neoplastic reactive cells in human effusions. *Acta Cytol.*, **33**, 491.

DERENZINI, M., PESSION, A. & TRERÈ, D. (1990). Quantity of nucleolar silver-stained proteins is related to proliferating activity in cancer cells. *Lab. Invest.*, **63**, 137.

DERVAN, P.A., GILMARTIN, L.G., LOFTUS, B.M. & CARNEY, D.N. (1989). Breast carcinoma kinetics. Argyrophilic nucleolar organizer region counts correlate with Ki67 scores. *Am. J. Clin. Pathol.*, **92**, 401.

EGAN, M.J., RAAFAT, F., CROCKER, J. & WILLIAMS, D. (1988*a*). Prognostic importance of nucleolar organiser regions in embryonal rhabdomyosarcoma. *J. Pathol.*, **154**, 477.

EGAN, M.J., RAAFAT, F., CROCKER, J. & WILLIAMS, D. (1988*b*). Comparative study of the degree of differentiation of neuroblastoma and mean numbers of nucleolar organiser regions. *J. Clin. Pathol.*, **41**, 527.

FLETCHER, G.H. (1973). Clinical dose-response curves of human malignant epithelial tumors. *Br. J. Radiol.*, **46**, 1.

GALL, J.G. & PARDUE, M.L. (1969). Formation and detection of RNA-DNA hybrid molecules in cytological preparations. *Proc. Natl Acad. Sci. USA*, **63**, 378.

GEHAN, E. (1965). A generalized Wilcoxon test for comparing arbitrarily singly-censored samples. *Biometrika*, **52**, 203.

GIRI, D.D., NOTTINGHAM, J.F., LAWRY, J., DUNDAS, S.A.C. & UNDERWOOD, J.C.E. (1989). Silver-binding nucleolar organizer regions (AgNORs) in benign and malignant breast lesions: correlation with ploidy and growth phase by DNA flow cytometry. *J. Path.*, **157**, 307.

GOLDSMITH, M.M., CRESSON, D.H. & ASKIN, F.B. (1987). The prognostic significance of stromal eosinophilia in head and neck cancer. *Otolaryngol. Head Neck Surg.*, **96**, 319.

GRIFFITHS, A.P., BUTLER, C.W., ROBERTS, P., DIXON, M.F. & QUIRKE, P. (1989). Silver-stained structures (AgNORs), their dependence on tissue fixation and absence of prognostic relevance in rectal adenocarcinoma. *J. Pathol.*, **159**, 121.

HALL, P.A., CROCKER, J., WATTS, A. & STANSFELD, A.G. (1988). A comparison of nucleolar organizer region staining and Ki-67 immunostaining in non-Hodgkin's lymphoma. *Histopathology*, **12**, 373.

HANSEN, A. & ÖSTERGÄRD, B. (1990). AgNOR counts in intraendometrial neoplasia. *J. Clin. Pathol.*, **43**, 518.

HERMANEK, P. & SOBIN, L.H. (1987). *TNM Classification of Malignant Tumours*. 4th ed. Springer-Verlag, Berlin, Heidelberg, New York, London, Paris, Tokyo.

HOWAT, A.J., GIRI, D.D., COTTON, D.W.K. & SLATER, D.N. (1989). Nucleolar organizer regions in Spitz nevi and malignant melanomas. *Cancer*, **63**, 474.

HSU, H.-C., CHEN, C.-L., HSA, M.-M., LYNN, T.-C., TU, S.-M. & HUANG, S.-C. (1987). Pathology of nasopharyngeal carcinoma. Proposal of new histologic classification correlated with prognosis. *Cancer*, **59**, 945.

HSU, S.M., RAINE, L. & FANGER, H. (1981). Use of avidin-biotin-peroxidase complex (ABC) in immunoperoxidase techniques: a comparison between ABC and unlabeled antibody (PAP) procedure. *J. Histochem. Cytochem.*, **29**, 577.

KAPLAN, E.L. & MEIER, P. (1958). Non-parametric estimation for incomplete observations. *J. Am. Stat. Assoc.*, **53**, 457.

MCNICOL, A.M., COLGAN, J., MCMEEKIN, W. & TEASDALE, G.M. (1989). Nucleolar organizer regions in pituitary adenomas. *Acta Neuropathol.*, **77**, 547.

MOORE, C., FLYNN, M.B. & GREENBERG, R.A. (1986). Evaluation of size in prognosis of oral cancer. *Cancer*, **58**, 158.

MURRAY, P.G., BOLDY, D.A.R., CROCKER, J. & AYRES, J.G. (1989). Sequential demonstration of antigens and AgNORs in frozen and paraffin sections. *J. Pathol.*, **159**, 169.

NAVONE, R., GASTALDI, M., FOTI, F., PIA, F. & BUSSOLATI, G. (1989). Immunoistochimica dei carcinomi faringei: correlazioni prognostiche. *Atti I Congresso Nazionale FISAPEC, Varese 24–27 maggio 1989*, p. 219 (Abstract).

NAIRN, E.R., CROCKER, J. & MCGOVERN, J. (1988). Limited value of AgNOR enumeration in the assessment of thyroid neoplasm. *J. Clin. Pathol.*, **41**, 1136.

ÖFNER, D., TÖTSCH, M., SANDBICHLER, P. & 4 others (1990). Silver stained nucleolar organizer region proteins (AgNORs) as a predictor of prognosis in colonic cancer. *J. Pathol.*, **162**, 43.

OOMS, E.C.M. & VELDHUIZEN, R.W. (1989). Argyrophilic proteins of the nucleolar organizer region in bladder-tumours. *Virch. Arch. A*, **414**, 365.

PICH, A., VALENTE, G., MARGARIA, E., AZZONI, L., TASSO, M. & STRAMIGNONI, A. (1991). Argyrophilic nucleolar organizer region counts and Ki67 scores in human renal cell carcinoma. *Path. Res. Pract.*, **187**, 482.

PLOTON, D., MENAGER, M., JEANNESSON, P., HIMBER, G., PIGEON, F. & ADNET, J.J. (1986). Improvement in the staining and in the visualisation of the argyrophilic proteins of the nucleolar organizer region at the optical level. *Histochem. J.*, **18**, 5.

RÜSCHOFF, J., PLATE, K., BITTINGER, A. & THOMAS, C. (1989). Nucleolar organizer regions (NORs). Basic concepts and practical application in tumor pathology. *Path. Res. Pract.*, **185**, 878.

RÜSCHOFF, J., BITTINGER, A., NEUMANN, K. & SCHMITZ-MOORMANN, P. (1990). Prognostic significance of Nucleolar Organizing Regions (NORs) in carcinomas of the sigmoid colon and rectum. *Path. Res. Pract.*, **186**, 85.

SAKO, K., PRADIER, R.N., MARCHETTA, F.C. & PICKREN, J.W. (1964). Fallibility of palpation in the diagnosis of metastases to cervical nodes. *Surg. Gynecol. Obstet.*, **118**, 989.

SHANMUGARATNAM, K. & SOBIN, L. (1978). Histologic typing of the upper respiratory tract tumors. *International histological type of tumours*, No. 19, WHO: Geneva.

SHANMUGARATNAM, K., CHAN, S.H., DE THE, G., GOH, J.E.H., KHOR, T.H., SIMONS, M.J. & TYE, C.Y. (1979). Histopathology of nasopharyngeal carcinoma. Correlations with epidemiology, survival rates, and other biological characteristics. *Cancer*, **44**, 1029.

SHIMOKAWARA, I., IMAMURA, M., YAMANAKA, N., ISHII, Y. & KIKUCHI, K. (1982). Identification of lymphocyte subpopulations in human breast cancer tissue and its significance: an immunoperoxidase study with anti-human T- and B-cell sera. *Cancer*, **49**, 1456.

SIVRIDIS, E. & SIMS, B. (1990). Nucleolar organizer regions: new prognostic variable in breast carcinomas. *J. Clin. Pathol.*, **43**, 390.

SMITH, R. & CROCKER, J. (1988). Evaluation of nucleolar region-associated proteins in breast malignancy. *Histopathology*, **12**, 113.

SURESH, U.R., CHAWNER, L., BUCKLEY, C.H. & FOX, A. (1990). Do AgNOR counts reflect cellular ploidy or cellular proliferation? A study of trophoblastic tissues. *J. Pathol.*, **160**, 213.

TAYLOR-PAPADIMITRIOU, J., PETERSON, J.A., ARKLIE, J., BURCHELL, J., CERIANI, R.L. & BODMER, W.F. (1981). Monoclonal antibodies to epithelium specific components of the human milk fat globule membrane: production and reaction with cells in culture. *Int. J. Cancer*, **28**, 17.

UNDERWOOD, J.C.E. (1974). Lymphoreticular infiltration in human tumours: prognostic and biological implications: a review. *Br. J. Cancer*, **30**, 538.

WALKER, R.A. (1988). The histopathological evaluation of nucleolar organizer region proteins. Commentary. *Histopathology*, **12**, 221.

WOLF, G.T., MAKUK, R.W. & BAKER, S.R. (1984). Predictive factors for tumor response to preoperative chemotherapy in patients with head and neck squamous cell carcinoma. *Cancer*, **54**, 2869.